# Mechanical and Physical Properties of Hydrophobized Lightweight Aggregate Concrete with Sewage Sludge

**DOI:** 10.3390/ma9050317

**Published:** 2016-04-27

**Authors:** Zbigniew Suchorab, Danuta Barnat-Hunek, Małgorzata Franus, Grzegorz Łagód

**Affiliations:** 1 Faculty of Environmental Engineering, Lublin University of Technology, 40B Nadbystrzycka Str., 20-618 Lublin, Poland; g.lagod@pollub.pl; 2 Faculty of Civil Engineering and Architecture, Lublin University of Technology, 40 Nadbystrzycka Str., 20-618 Lublin, Poland; d.barnat-hunek@pollub.pl (D.B.-H.); m.franus@pollub.pl (M.F.)

**Keywords:** sewage sludge, lightweight-concrete, hydrophobization

## Abstract

This article is focused on lightweight aggregate-concrete modified by municipal sewage sludge and lightweight aggregate-concrete obtained from light aggregates. The article presents laboratory examinations of material physical parameters. Water absorptivity of the examined material was decreased by the admixture of water emulsion of reactive polysiloxanes. Water transport properties were determined using Time Domain Reflectometry, an indirect technique for moisture detection in porous media. Together with basic physical parameters, the heat conductivity coefficient λ was determined for both types of lightweight aggregate-concrete. Analysis of moisture and heat properties of the examined materials confirmed the usefulness of light aggregates supplemented with sewage sludge for prospective production.

## 1. Introduction

Energetic modernization of exploited domestic resources has become a major economic activity in the recent years. Its importance is underlined by the tendency to minimize the primary energy consumption [1]. Introduction of the UE 2006/32/WE3 Directive on 17 May 2006 imposes an obligation on Poland to undertake special activities in order to reduce final energy consumption by users of buildings within the consecutive nine years starting from 1 January 2008. To improve the energetic performance of the building industry, promotion of application of renewable sources of energy to power buildings and employment of energy saving technologies in construction of buildings was assumed to be preferential [2].

The analysis of the Polish Central Statistical Office data (1997–2007) has confirmed that the final energy consumption in Polish households is mainly attributed to central heating, accounting for 31%–71% of energy consumption [1], which means that the average is similar to the final energy consumption in Europe, equal to 50% [3,4]. The high energy consumption by the housing sector results in emission of large amounts of carbon dioxide into the atmosphere, which accounts for *ca.* 50% of the total emission of gases. The effect of building technologies on the environment, mainly fuel consumption during exploitation and environment pollution due to CO_2_ emission, is frequently mentioned in the literature [5,6,7,8,9].

Physical and moisture properties of building materials are the main factors affecting air quality, heat comfort, and energy consumption by buildings, as well as durability [10,11]. In non-insulated buildings, the phenomenon of condensation occurs, especially due to improper thermal insulation and ventilation of rooms [12]. This is mainly important for partitions touching the ground, where capillary water transport essentially affects heat flow by a 4- to 6-fold increase in heat conductivity of porous materials, which was confirmed in literature [13,14]. Water present in masonry negatively influences indoor air, creating suitable conditions for harmful microorganisms to develop and biological and chemical corrosion, thereby increasing exploitation costs. Moisture and temperature are the most important parameters that influence development of mold and fungi in building barriers [15,16]. Improper, changeable moisture and temperature conditions contribute to the growth of mold, which was confirmed by the results of laboratory experiments [15,17,18]. An increase in moisture also results in changes in indoor microclimate and a decrease in thermal comfort, which may lead to disorders of the respiratory system, infections, allergies, and eye or skin irritation [19].

Production of ecological and energy-saving building materials becomes a common technology aimed at improving energetic effectiveness of buildings in accordance with the European Union Directive 2006/32/WE3 [2]. One of the materials applied for the energy-saving civil engineering is lightweight aggregate-concrete, especially because of its heat and moisture parameters. Compared to traditional concrete, the lightweight aggregate-concrete facilitates reduction of the weight of construction elements. Most natural aggregates have a particle density between 2.4 and 2.8 g/cm^3^, typically 2.6 g/cm^3^, while lightweight aggregates have a particle density between 0.8 and 2.0 g/cm^3^ [20].

The decrease in dead weight could lead to reduced construction costs, since it can decrease the size of the foundation and structural elements such as columns or walls.

To obtain lightweight concrete, lightweight aggregates modified with municipal sewage sludge could be applied, which was confirmed by the results of scientific research [21,22].

The reutilization of industrial wastes and the use of recycled materials in construction applications have been a common practice and have increased worldwide over the last decades [23,24]. Heat treatment can convert some types of wastes into ceramic products [25,26].

Due to the increase in the number of Sewage Treatment Plants and the efficiency of sewage treatment processes with a reduction of carbon compounds and biogens, the amount of emerging sewage sludge significantly increases [27]. Sewage sludge often contains heavy metals, which are not sanitary safe after stabilizing through the process of methane digestion [28]. In many cases, sewage sludge is also dangerous to the natural environment and, therefore, it ought to be suitably processed. Regulations and acts imposed by the European Union limit the sewage sludge deposition in landfills and its reuse in agriculture [29,30,31,32,33,34,35]. One of the methods for utilization of sewage sludge is to apply it in production of ceramic materials [36,37] and energy-saving lightweight aggregate-concrete blocks [20,21]. Unfortunately, sewage sludge is often characterized by high moisture absorptivity due to the light aggregates structure. It causes a serious problem in the composition of the lightweight aggregate-concrete mixtures and in the ready-products. It essentially affects the heat flow process by an increase in the heat conductivity of the materials. The type and distribution of pore networks as well as their connection with the aggregate surface is an important feature for production of lightweight concretes [38]. Differences between volumetric densities of the lightweight aggregate and covering it with cement mortar causes the aggregates to flow out in cases when the cement mortar has no suitable viscosity. To avoid the unfavorable phenomenon of subtraction of water required for the hydration process by lightweight aggregate, several procedures can be conducted. One of the methods is initial wetting to protect the aggregates from autogenic contraction [39]. Another solution is to cover the aggregates with cement grout or ceramic shell, which provides lower water absorptivity of the aggregates, increases the density of aggregate particles and, thus, essentially influences concrete strength [38,40,41].

A new technology is impregnation of aggregates, which closes air gaps preventing water penetration with constant adherence of particles to the cement matrix [29,39]. Hydrophobization of the aggregates and mortar decreases capillary water absorption, but still does not seal the pores or capillaries, which enables free vapor permeability [42,43,44,45,46]. Water-soluble organic silica compounds, *i.e.*, siloxanes, can be used as hydrophobizing agents in the amount of 1%–2% in relation to cement mass [46,47,48,49]. The use of hydrophobization during lightweight block manufacture would eliminate the phenomenon of excessive moisture during exploitation of building objects. Additionally, it would offer considerable protection against transport of saline solutions into the brickwork, which would cause material destruction due to multiple processes of freezing and defrosting during wintertime and the phenomenon of dissolved salt crystallization. In addition, materials containing salt are characterized by higher moisture than materials that are free from salts [50]. Excessive moisture significantly influences the heat flow process by an increase in heat conductivity of the materials and thus losses of energy [13,51,52].

The results of the present study can possibly be used to establish guidelines for practical applications of lightweight aggregates concrete with sewage sludge foamed by hydrophobic agents, which has slightly different characteristics compared to those of traditional lightweight concrete. The analysis of heat-moisture as well as physical and mechanical properties of concrete will confirm the usefulness of lightweight aggregates with sewage sludge addition for further production of energy-saving and ecological lightweight blocks. Introduction of new technologies of energy-saving materials in the building industry provides greater and significant potential, which would lead to a decrease in final energy consumption in the building and housing sector.

## 2. Materials and Methods

The presented research consisted of three stages of examinations: determination of parameters of raw materials used for preparation of aggregates, determination of characteristics of aggregates, and finally (and mainly) determination of the parameters of lightweight aggregate concretes.

### 2.1. Determination of the Characteristics of Raw Materials Used in the Production of Lightweight Aggregates

Clay for aggregate production was taken from “Budy Mszczonowskie” bed, Poland, which is currently being exploited by the Light Aggregates Company “Keramzyt”. Sewage sludge was taken from the municipal sewage treatment plant “Hajdów” in Lublin, Poland, which is a mechanical-biological plant purifying municipal and partially industrial sewage. Sewage sludge was sampled from the mechanical dewatering station. The physical-chemical properties of the sewage sludge were determined based on the following regulations [53,54,55,56]. The chemical composition of sewage sludge was determined using Atomic Emission Spectroscopy (ICP), JARRELL ASH Enviro and inductively coupled plasma mass spectrometry (ICP/MS), Perkin Elmer Elan 6000.

The chemical composition of clay was determined by the X-ray fluorescence (XRF) method. The Philips spectrometer PW 1404 (Panalytical, Almelo, The Netherlands) was applied. The induction source was constituted by a lamp with a double anode (Cr-Au) with maximum power of approximately 3 kW.

The mineral composition of all samples (clay, sewage sludge) was determined by X-ray diffraction (XRD) using X’pert PROMPD (Panalytical) with a PW 3050/60 goniometer (Panalytical), a Cu lamp, and a graphite monochromator. The analysis was performed within the angle range of 5°–65° (2 Theta). Philips X’Pert Highscore software (High Score Plus v. 4.1) was used to process the diffraction data. The identification of mineral phases was based on the PDF-2 release 2010 database formalized by the ICDD. Weight of the samples for XRD and XRF examinations was 4 g and each measurement was conducted in 3 replications.

The morphological forms and the chemical composition of substrates and products were determined by means of a scanning electron microscope (SEM) FEI Quanta 250 FEG (FEI, Hilsboro, OR, USA) equipped with a system of chemical composition analysis based on energy dispersive spectrometry (EDS) X-ray-EDS from EDAX company (EDAX Inc., Mahwah, NJ, USA). Investigated area of the samples for SEM analyses was *ca.* 25 mm^2^.

### 2.2. Manufacturing of Lightweight Aggregates

The clay was dried to a constant mass directly after sampling. Then, it was mixed in a ball mill to a fraction of <0.5 mm. Sewage sludge was dried at a temperature of 105 °C to a constant mass, then ground in the mortar grinder to a fraction below 1.0 mm. Dried sludge was ground and then the mixture was prepared in the following proportions: 10% by weight of sludge, 90% by weight of clay. The process of making the substance homogeneous consisted of mixing the components with the corresponding portion of water to achieve plastic consistency. Then, the formed spheres of an 8 to 16 mm coarse fraction were dried to achieve air-dry state and kept in a laboratory oven at 110 °C for 2 h. Dried samples were transferred to a chamber furnace and fired at 1150 °C for 30 min. The second type of aggregates came from the Lightweight Aggregates Company “Keramzyt” in Mszczonów, Poland.

### 2.3. Determination of the Properties of the Lightweight Aggregates

The aggregate obtained with the above-mentioned method was tested in order to determine its physical and mechanical characteristics according to the current standards and regulations. Minimal mass of material applied for the measured samples was at least 1 kg. Loss of bulk density (ρ_b_, expressed in g/cm^3^) of the lightweight aggregate was determined using the following standard [57]. Particle density (apparent ρ_a_ and dry ρ_d_, expressed in g/cm^3^) and water absorptivity after 24 h (WA_24h_) of aggregate immersion was determined using a procedure described by the standards [58].

Based on the data obtained, porosity of the lightweight aggregates was determined [57]. Compressive strength was determined according to the standard [59]. The examination consisted in determination of percentage mass loss of the aggregate partitions due to compression, which was used to calculate the resistance to crushing *X*_r_:

*X*_r_ = *m* − *m*_1_/*m* × 100 (1)

where *m* is the mass of the sample before testing (g), and *m*_1_ is the mass of the sample after examination (g).

Frost resistance was determined according to the standard [60]. The aggregate sample was moistened and frozen/defrosted in 10 cycles in the temperatures of −17.5 °C and *ca.* 20 °C.

The Maximum Leaching (ML) method was applied to achieve the immobilization level of heavy metals from building materials [61,62]. It allowed determination of the maximal leaching of the substances from the tested materials in extreme conditions of the environment. 500 mL of distilled water acidified with 1 n HNO_3_ to pH = 4 was added to 5 g of ground-up samples, which had a fraction below 125 µm. Samples prepared in this way were shaken for 5 h, and pH of the solution throughout the process was kept constant using 1 n HNO_3_. After leaching, the suspensions were centrifuged. The clear solutions obtained were decanted and the concentrations of heavy metals were detected with the ICP-MS method using a ICP-OES (Jobin Ywon U-238) spectrometer (HORIBA Jobin Yvon, Edison, NJ, USA).

### 2.4. Manufacturing of Lightweight Concrete

To prepare the samples of concrete, CEM I 32.5R cement, light aggregates described above (8–16 mm), quartzite sand (0–2 mm) from Suwalskie Kopalnie Surowców Mineralnych, Suwałki, Poland, and water from the municipal water supply system were used. Water solvable organic silica compounds, polysiloxanes, in an amount of 2%, compared to the cement mass, were used as a hydrophobic material. The concrete samples were made according to the Polish standards [63,64]. The assumptions to the design included plastic consistency *D*_max_ = 16 mm. The composition of the examined light concretes per 1 m^3^ is presented in Table 1.

The dimensions of the samples were 150 mm × 150 mm × 150 mm. They were formed directly after the concrete compounds had been mixed. They were condensed in two layers with the help of vibrations until the cement grout appeared at the mortar surface. The samples were disassembled after 24 h of maturation and placed in a water basin until full average strength was reached according to the standard PN-EN 206-1:2003/A2:2006P [63].

### 2.5. Determination of Lightweight Concrete Properties

Density and porosity were determined using the standard PN-EN 1936:2010 [65]—a method with a pycnometer, in laboratory conditions, at a temperature of 20 °C. Apparent density determination was conducted after 28 days of concrete maturation. To determine apparent density, 6 cuboid concrete samples (150 mm × 150 mm × 150 mm) were prepared. Apparent density and open porosity volume were determined using the standard [66].

**Figure 1:**
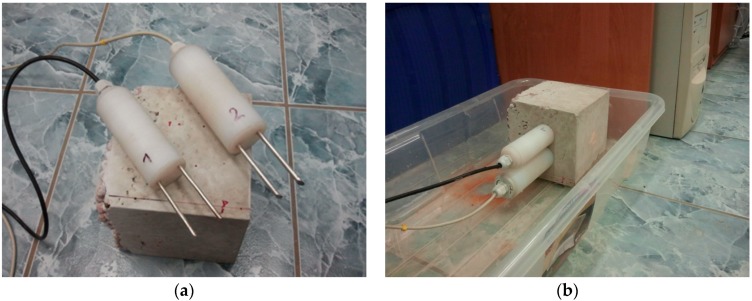
Determination of the capillary uptake process: (**a**) presentation of the sample and the modified probes; and (**b**) measuring setup ready for the experiment.

Examination of the physical parameters of the lightweight concrete blocks was supplemented with determination of the capillary rise capability of both types of materials. The research was conducted using the TDR (Time Domain Reflectometry) technique, described in the following papers [67,68,69,70,71]. For the experiment, three sets of samples of each concrete type were prepared. Before the examination, the samples were dried to a constant mass. The samples had the following dimensions: 150 mm × 150 mm × 150 mm. TDR probes used in the experiment were of our own construction, intentionally developed for moisture measurement in hard building materials. They were installed in the material samples in the following positions: 5 and 10 cm above the constant water level (Figure 1). The TDR multimeter used in the experiment was produced by E-Test, Lublin, Poland. The standard uncertainty of the measurement of the applied setup according to our own test is *ca.* 1.5 vol %. The research was conducted at a constant temperature of 20 ± 1 °C for 350 h with constant monitoring of dielectric permittivity changes, recalculated into moisture according to the formulas presented in the following paper [72].

To determine the heat conductivity coefficient, a Laser Comp FOX 314 plate apparatus (TA Instruments, New Castle, DE, USA) was used. For that purpose, 3 plates of each concrete type were prepared. Each plate had the following dimensions: 300 mm × 300 mm × 50 mm. The research was conducted for dry samples and 3% moist, obtained by storage of the material in a room with relative humidity equal to 70% for 4 weeks. For determination of the heat conductivity coefficient of lightweight concretes, two temperatures were applied: 20 °C for the heating plate and 0 °C for the cooling plate. The average temperature was 10 °C. According to the producer, the absolute thermal conductivity accuracy of the device is ±2% and reproducibility ±0.5%.

Examination of compressive strength was conducted according to the standard [73]. For the experiment, 6 cubic lightweight concrete samples (150 mm × 150 mm × 150 mm) were applied. Evaluation of the grade of the concrete was performed using a Controls compression tester after 28 days of maturation, when the samples gained average compressive strength.

Compressive strength was calculated using the following formula:

ƒ’_ck_ = *F*/*A*_c_ (2)

where ƒ’_ck_ is compressive strength (MPa); *F* is maximal load at destruction (N); and *A*_c_ is sample cross section area under compressive force, calculated with the declared dimension (mm^2^) according to the standard [70].

Examinations of frost resistance were conducted using the direct method [63]. Six concrete samples were exposed to 25 cycles of freezing–defrosting processes according to the standard [63].

The direct method involves cyclic freezing and defrosting of the samples in air with a temperature of −18 ± 2 °C for at least 4 h, and then defrosting in water at a temperature of 18 ± 2 °C for 2 to 4 h. After the final cycle, the loss of weight and pressure strength is estimated. Each step of freezing and defrosting is a single cycle of the research. After the final step, the samples were dried out and weighed. With the mass readouts obtained, the percentage mass loss “*S*” was determined. Then the compressive strength was tested and its percentage loss was determined compared to the samples without the freezing–defrosting procedure.

The percentage mass loss of the particular samples was determined using the following formula:

(3) $$S=\frac{m_{0}-m_{n}}{m_{n}}\times 100\%$$

where *S* is percentage mass loss for the particular samples (%); *m*_0_ is sample mass before the freezing–defrosting procedure (g); and *m*_n_ is sample mass after the freezing–defrosting procedure (g).

## 3. Results

Data obtained in the whole experiment were divided into three groups: (i) parameters of raw materials used for aggregate preparation; (ii) characteristics of aggregates; and (iii) parameters of lightweight aggregate concretes. The tested material for production of lightweight aggregates was sewage sludge taken from the municipal sewage treatment plant “Hajdów” in Lublin and clay from “Budy Mszczonowskie” bed in Poland.

### 3.1. Physical-Chemical Characteristics of Raw Materials

**Figure 2:**
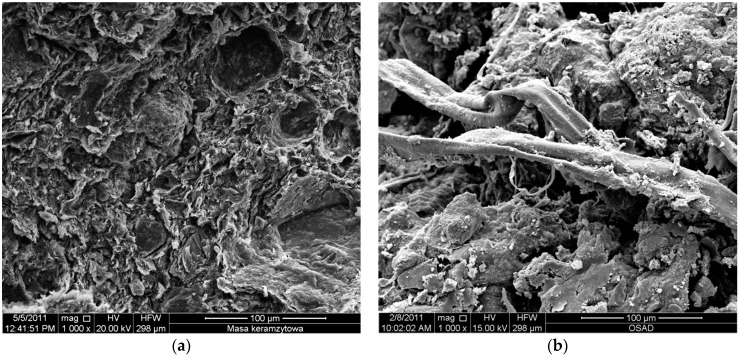
SEM micrographs of: (**a**) clay; and (**b**) sewage sludge.

Test results of the examination of sewage sludge from the sewage treatment plant “Hajdów” in Lublin are presented in Table 2. The research confirms the increased presence of zinc and copper ions. Smaller amounts of chromium, nickel, lead, and cadmium ions were also detected. Figure 2 presents SEM micrographs of clay and sewage sludge.

### 3.2. Physical and Mechanical Properties of Lightweight Aggregates Modified with Sewage Sludge

Physical and mechanical properties of the lightweight aggregate modified with sewage sludge are presented in Table 3. Data of leachability measurements of chromium, cadmium, copper, nickel, lead, and zinc in water extracts are presented in Table 4.

**Figure 3:**
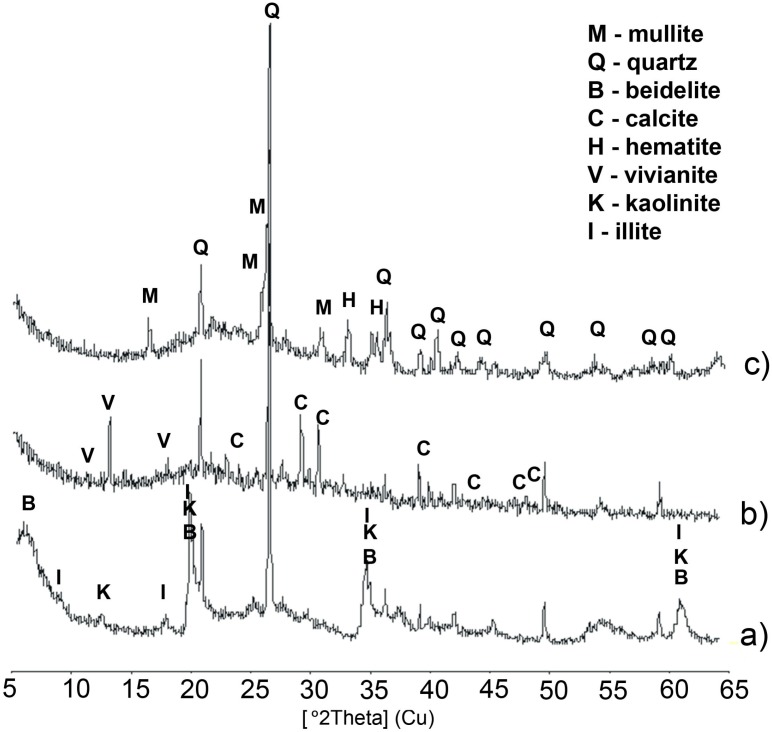
XRD patterns of: (**a**) Mszczonów clay; (**b**) sewage sludge; and (**c**) lightweight aggregates burnt at a temperature of 1150 °C.

**Figure 4:**
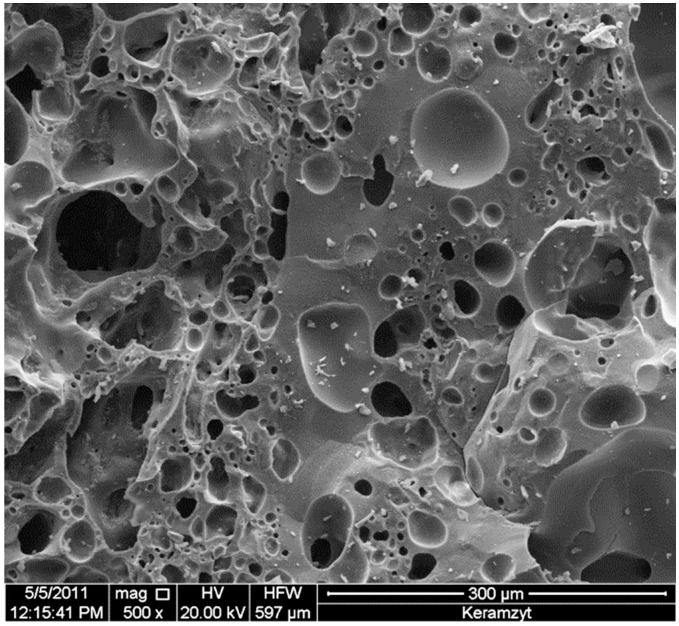
Scanning microscope image of the structure of lightweight aggregates.

With X-ray diffraction analysis, the inorganic crystalline composition of the lightweight aggregate was established. Angular positions and intensity of diffraction reflections are presented in Figure 3. Figure 4 presents the porous structure of the aggregate observed under a scanning microscope.

### 3.3. Parameters of Lightweight Aggregate-Concretes

**Figure 5:**
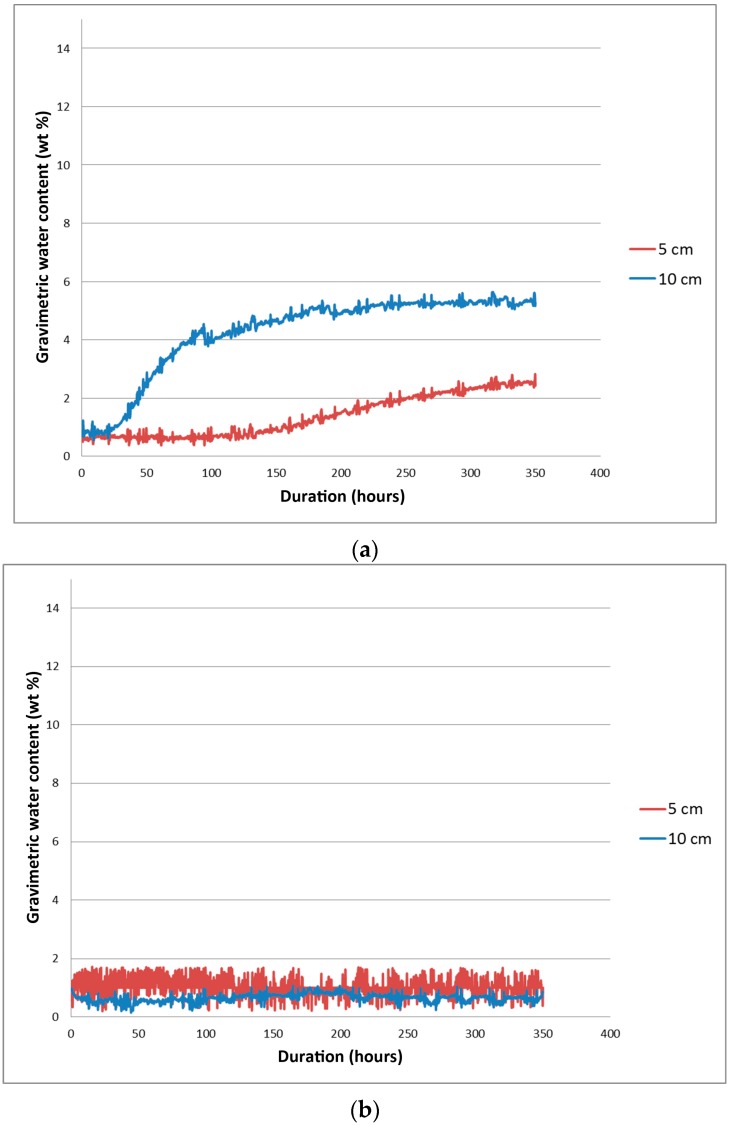
Capillary rise phenomenon determined in this research: (**a**) lightweight aggregate concrete with aggregates from Mszczonów; and (**b**) lightweight aggregate concrete with aggregates from sewage sludge.

The physical properties of the examined lightweight concretes are shown in Table 5. Readouts of apparent permittivity changes in time obtained using Time Domain Reflectometry were recalculated into moisture and presented in the form of diagrams in Figure 5.

**Figure 6:**
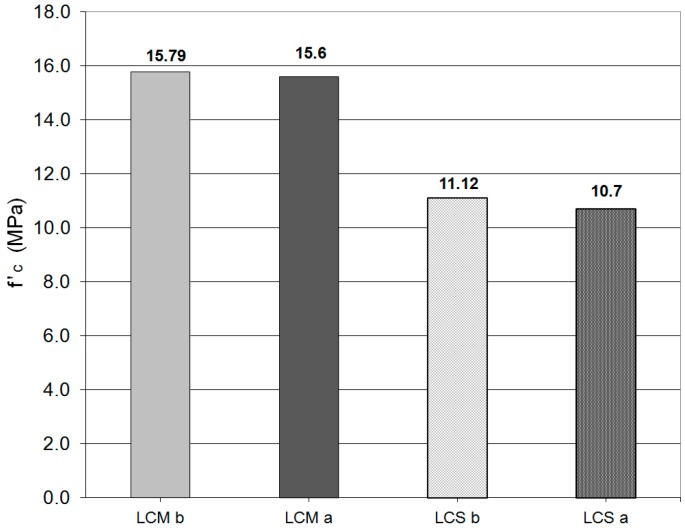
Compressive strength of samples examined for frost resistance-comparative samples and frozen samples: LCM b—lightweight concrete “Mszczonów” before freezing; LCM a—lightweight concrete “Mszczonów” after freezing; and LCS b/a—lightweight concrete sewage before/after freezing.

The determined values of heat conductivity coefficients λ (average of three samples) are presented in Table 6. Table 7 presents strength parameters of the examined lightweight concretes and Figure 6 presents data of compressive strength of the samples examined for frost resistance.

**Figure 7:**
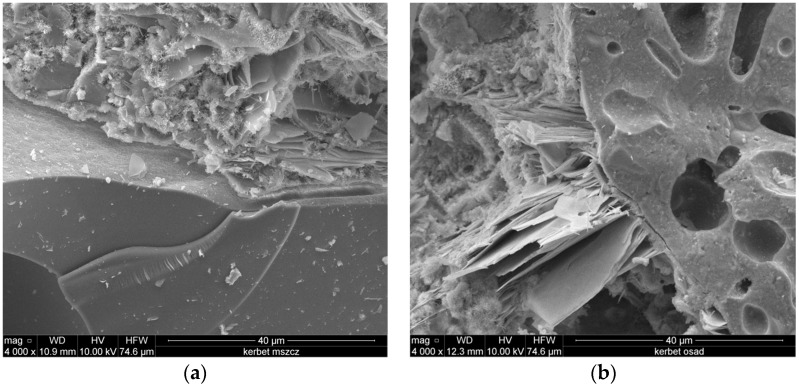
SEM investigation in contact point between: (**a**) lightweight aggregates from Mszczonów and cement mortar; and (**b**) lightweight aggregates with sewage sludge and cement mortar.

**Figure 8:**
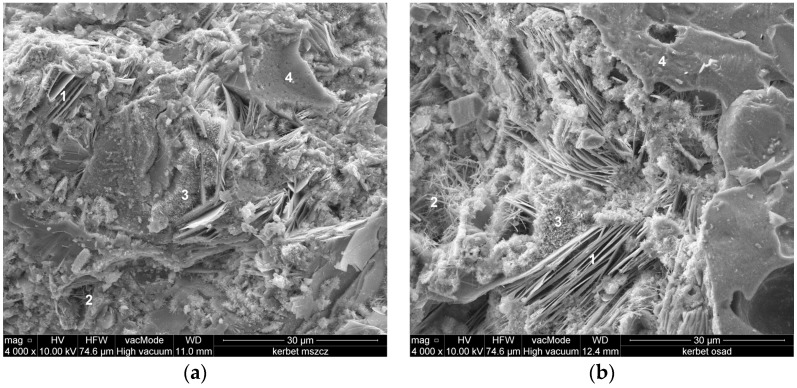
Lightweight aggregate concrete with aggregates: (**a**) from Mszczonów; and (**b**) from sewage sludge-mineral phases in cement mortar. 1—Portlandite; 2—Etryngite; 3—C–S–H phase; honeycomb type; 4—C–S–H phase, solid type.

**Figure 9:**
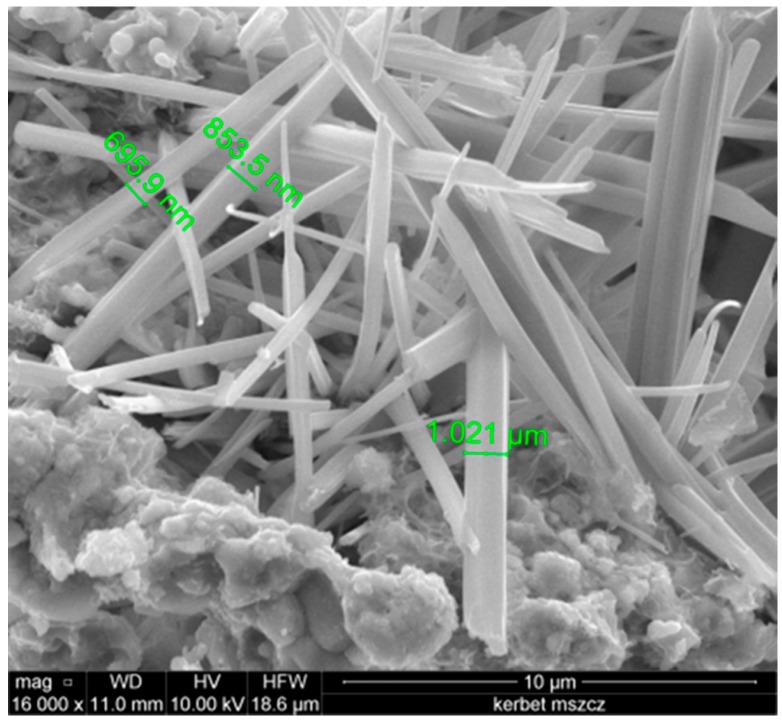
Needle-shaped etryngite.

Figure 7 shows the form and morphology of the crystalline phases formed in lightweight concrete observed under the scanning microscope. Figure 8 and Figure 9 present scanning microscope photographs of lightweight aggregate concrete with aggregates from Mszczonów and those manufactured from sewage sludge, respectively.

## 4. Discussion

### 4.1. Characteristics of Raw Materials

The data presented in Table 2 show the characteristics of raw materials used for preparation of the samples. In the chemical composition of clay, the presence of SiO_2_ (71.3%), Al_2_O_3_ (14.8%), Fe_2_O_3_ (7.3%), MgO (2.6%), K_2_O (1.44%), Na_2_O (0.95%), CaO (0.65%), TiO_2_ (0.56%) BaO (0.23%), MnO (0.11%), and ZnO (0.06%) were detected. X-ray diffraction analysis was performed to establish the inorganic crystalline composition of the sludge and clay from Mszczonów. The X-ray diffraction photograph of the sample phase shows angular positions and intensity of diffraction reflections. Mineral phases were recognized by the characteristic interlayer distances. The main mineral components of sewage sludge were identified: calcite (*d* = 3.86, 3.04, 2.28, 1.91 Å), vivianite (*d* = 7.94, 6.70, 4.90 Å), and quartz (*d* = 4.27, 3.34, 2.45, 1.82 Å). The main mineral components of clay from “Budy Mszczonowskie” bed are clay minerals represented by beidelite (*d* = 15.15, 4.44, 3.02, 2.59, 2.48, 1.49 Å), illite (*d* = 10.01, 5.02, 4.48, 3.34, 2.59, 1.49 Å), and kaolinite (*d* = 7.14, 4.48, 4.36 Å), which are accompanied by quartz (*d* = 4.27, 3.34, 2.45, 1.82 Å) in minor amounts.

The SEM analysis proved the presence of aggregates of loamy minerals (Figure 2). They are present in the form of thin grains with a husk-shaped cross-section with the dimensions of 10 µm (Figure 2a). The structure of sewage sludge with visible accumulation of phosphorous minerals is presented in Figure 2b.

### 4.2. Properties of Lightweight Aggregates

In Table 3, physical and mechanical properties of the lightweight aggregate modified with sewage sludge are presented.

The results obtained showed that the dry particle density was 2580 kg/m^3^, apparent density 810 kg/m^3^, and the bulk density 410 kg/m^3^. These values are characteristic for lightweight aggregates, which should be less than 2000 kg/m^3^ in apparent particle density values according to the standard [74]. Therefore, the artificial aggregates manufactured in this study were classified as lightweight aggregates.

The water absorptivity of the aggregates reaches 15.9% and is probably a consequence of the open porosity of the grains and heterogeneity of the grain shape. The saturation obtained is comparable to water absorptiveness of the commercial aggregates Lytag, which is equal to 17.55%. The apparent particle density of the Lytag aggregates is equal to 1340 kg/m^3^ and bulk density-730 kg/m^3^ [75]. The parameters obtained are slightly higher than the parameters of lightweight aggregates with sewage sludge.

The absorptivity of Aardelite aggregates produced from industrial waste and carbon dioxide is 21.2% and the apparent particle density is slightly higher and reaches the value of 2100 kg/m^3^ [76]. The generally acceptable water absorption capacity based on 24-h soaking in water is lower than 20%. Therefore, lightweight aggregates produced with sewage sludge fulfill the above requirements. The resistance to crushing of the examined aggregates is 34.7%, and its relatively low value is probably a consequence of the high porosity of the aggregates and the presence of a glassy layer both inside the aggregates and at their surface, which leads to a decrease in the material strength.

Frost resistance does not exceed 1.0%. After examination, the aggregate grains do not show any cracks. Lightweight aggregates can be treated as the F1 category because the percentage mass loss is lower than the required maximum 1% for this category, according to the standard [77]. Hence, they fulfill the frost resistance requirements.

The aggregate porosity is 68%. This value provides suitable moisture absorptivity, high water vapor diffusivity, but also suitable thermal insulation. With its advantages, the lightweight aggregate obtained should be successfully used in energy-saving construction, for example in production of lightweight concretes and wall blocks, as well as thermal insulation for floors or roofs.

The leachability measurements of chromium, cadmium, copper, nickel, lead, and zinc in water extracts showed that the presence of metals is much lower than the tolerable values (Table 3). This means that it is possible to use the tested aggregate with sewage sludge as a valuable ecological building material. Aggregates supplemented with sewage sludge additives, burnt at the temperature of 1150 °C, permanently bind the cations of heavy metals inside their structure.

In the mineral compound of the lightweight aggregate burnt at the temperature of 1150 °C, the glassy phase is present and is recognized by the characteristic rise in the background of the diffractogram within the angular extent 15°–30° (2 Theta) and mullite (*d* = 5.41, 3.43, 3.39, 2.88 Å). The mineral composition of the aggregates is supplemented by hematite (*d* = 2.70, 2.51 Å).

Figure 4 proves that the pores are strongly dissimilar in their shape and dimensions with domination of pores with round shapes occasionally accompanied by pores of irregular shapes. Smaller round shaped pores reach the dimensions of 10 µm and the dimensions of the greater pores vary between 30 and 100 µm. On the internal surface of the pores, the presence of the glassy phase covering them is clearly seen.

### 4.3. Characteristics of Lightweight Aggregate-Concretes

This section contains the analysis of physical-chemical parameters of the aggregate-concretes obtained from Mszczonów and manufactured from sewage sludge.

As shown by the above-mentioned measurements and calculations, the real mass of the concrete unit (apparent density) is ρ_p_ = 1344–1442 kg/m^3^. It is typical for light concretes, with the apparent density not exceeding 800–2000 kg/m^3^. In addition, the dry particle density was determined with a pycnometer to be ρ = 2376 and 2450 kg/m^3^. The percentage volume of pores in the concrete with sewage sludge reaches 45.1 vol % and is 12.86% higher than the light concrete with the aggregates from Mszczonów. According to the standard [63], both concretes have the density class D1.6.

The conducted research of the capillary rise using the TDR method confirmed the decreased capillary parameters of the examined materials. In the case of the sample with aggregates from Mszczonów (Figure 5a), the progress of the process was very slow. The first visible water presence readouts were noticed at a lower probe (5 cm above the water level) after 30 h of the experiment. The maximum water content was reached after *ca.* 250 h and was below 6 wt %, which was close to material absorptivity (7%). Afterwards, no progress in water uptake was noticed. The second probe, which was placed 10 cm above the water level, revealed lower moisture levels, delayed in time.

In the case of the sample with aggregates with sewage sludge, no significant water increase was noticed.

The heat conductivity coefficient λ of the lightweight aggregate-concrete with aggregates from Mszczonów in a dry state is *ca.* 7% lower compared to moist material (3% of moisture) and 10% higher compared to concrete with sewage sludge. The difference between both moist concrete types (3% of moisture) is *ca.* 19.44%, which confirms better thermal properties of the lightweight aggregate-concrete with sewage sludge.

The average compressive strength of concrete with aggregates from Mszczonów was 15.79 MPa. According to [63], this concrete is classified as class LC 12/13. Average compressive strength of concrete with aggregates made of sewage sludge is *ca.* 29.5% lower than that of aggregate without sludge additives. The lightweight concrete represents class LC 8/9. None of the investigated samples showed strength diverting more than 10% from the average results. That is why all samples were considered in the calculations of the average value. Sewage sludge additives caused a decrease in compressive strength, which resulted in lower load capacity of lightweight aggregate-concrete blocks.

During the examination of frost resistance, the average mass loss of all samples did not exceed 1%. For the concretes with sewage sludge additives, it reached 0.9% and in the case of concretes without sludge additives, it was 0.2%. Significant differences were noticed for pressure strength of the frost samples compared to the reference samples (Figure 6).

The lowest compressive strength decrease, not exceeding 5% (acceptable mass loss in frost resistance [63]), was observed in the case of both lightweight-concretes. It should be underlined that in the case of the lightweight concrete from Mszczonów, the differences were *ca.* 0% (within the measurement error). In the case of the lightweight concrete with sewage sludge, the differences reached the acceptable value of the strength decrease—*ca.* 5% [63].

The scanning microscope photographs show the form and morphology of the crystalline phases formed the in lightweight concrete. Hardened cement mortar obtained from Portland cement consists mainly of 70% of hydrated calcium silicates, so-called C–S–H phases and about 30% of calcium hydroxide and products aluminate hydration and calcium aluminate-ferrate. The microscopic research confirmed good adhesion in contact points between the lightweight aggregates and the cement mortar. No scratches, cracks, or empty gaps were noticed in the above-mentioned contact points (Figure 7).

Two characteristic mineral phases are seen in the cement mortar. The first one is formed by the C–S–H phase, whose morphology resembles a three-dimensional structure of the honeycomb type (Figure 8) and tightly compacted agglomerates of the amorphous phase C–S–H. Other ingredients are the hexagonal, massive, tabular crystals of portlandite forming concretions. These tabular accumulations dominate in dimensions (10 μm) over the C–S–H phase. In both cases, small amounts of needle shaped ettringite crystals are present in the cement mortar of lightweight-concretes (Figure 9).

## 5. Conclusions

Using lightweight aggregate-concrete modified with sewage sludge, it is possible to manufacture lightweight concrete with a density in the range of 1400–1960 kg/m^3^.

Additives of sewage sludge caused a decrease in the apparent density by *ca.* 7% and an increase in the total porosity of the concrete by 12.86%, compared to the concrete from lightweight aggregate available on the building market. This was also confirmed by SEM verifications. The microscopic research of contact points between lightweight aggregates and cement mortar proved good adhesion. No scratches, cracks, or empty gaps were noticed in the above-mentioned contact points.

Absorptivity of both lightweight aggregate-concretes is at a low level, between 3% and 7%, due to the application of the hydrophobic preparation as a concrete additive. It must be underlined that the lightweight aggregates with sewage sludge, due to higher absorptivity and porosity, absorbed more impregnate, decreasing the total absorptivity of the light concrete by *ca.* 57%. The TDR research of the capillary rise process proved total inhibition thereof.

The sewage sludge additives in lightweight aggregate-concretes decreased the heat conductivity coefficient λ by *ca.* 7%–10%.

The sewage sludge additives caused a decrease in the compressive strength, which resulted in lower load capacity of lightweight aggregate-concrete blocks.

The addition of sewage in lightweight aggregates production caused a slight decrease (by *ca.* 4%) in frost resistance of the examined concretes but still at an acceptable level.

The application of sewage sludge presented in this article is a good perspective for utilization of the increasing sewage sludge quantities resulting from the process of wastewater treatment in Sewage Treatment Plants.

The presented examinations have proven that sewage sludge can be applied as an additive for lightweight aggregate production, which then can be successfully applied for the production of light concretes. Importantly, sewage sludge improves the thermal parameters of lightweight aggregate-concretes, which in turn can increase the energetic performance of the buildings, which are constructed with the use thereof. Unfortunately, the application of sewage sludge decreases the compressive strength, which is not favorable for load-bearing walls of the buildings. Lightweight aggregate-concrete supplemented with sewage sludge, produced, and analyzed in this paper can be successfully applied for production of elements filling wooden structures or in the form of blocks for curtain walls of buildings.

## Figures and Tables

**Table 1:** Composition of light concretes applied per 1 m^3^ (kg).

Type of Concrete	Cement (C)	Sand (F)	Aggregates (G8-16)	Water (W)	Hydrophobic Additive (H)
With aggregates from Mszczonów	280	470	540	225	5.6
With aggregates from sewage sludge	272	453	499	202.5	5.44

**Table 2:** Physical-chemical characteristics of sewage sludge from the Sewage Treatment Plant “Hajdów”, Lublin.

Physico-Chemical Characteristics	Average Values
Moisture content (%)	78.3
Alkalinity (mgCaCO_3_/L)	740
Total organic matter (%)	64
pH	7.42
Chemical oxygen demand (mg/L)	132,586
Volatile fatty acids (mg/L)	92
Dry mass (%)	18.87
Loss on ignition (%)	59.23
Residue on ignition (%)	38.42
Density (g/mL)	0.734
Zn(II) (ppm)	210.76
Cu(II) (ppm)	82.74
Cr(II) (ppm)	12.97
Ni(II) (ppm)	6.23
Pb(II) (ppm)	7.94
Cd(II) (ppm)	3.45
Phosphorous (mg/L)	125

**Table 3:** Physical and mechanical properties of the lightweight aggregate burnt at a temperature of 1150 °C.

Parameter	Lightweight Aggregate (1150 °C)
Dry particle density, ρ_d_ (kg/m^3^)	2580
Apparent particle density, ρ_a_ (kg/m^3^)	810
Bulk density, ρ_b_ (kg/m^3^)	410
Water absorptivity (%)	15.9
Porosity (%)	68
Frost resistance (%)	0.9
Resistance to crushing, *X*_r_ (%)	34.7

**Table 4:** Leaching of heavy metals from water extracts.

Method of Analysis	Leaching Content (mg/L)					
	Cu	Zn	Cd	Cr	Ni	Pb
Maximum Leaching Method	0.19	0.12	0.04	0.07	0.05	0.02
Admissible values ^1^	<2.0	<2.0	<0.05	<0.5	<0.1	<0.1

^1^ The most restrictive limits of concentrations for industrial sludge, Polish Standard Requirements according to the Regulation of the Minister of the Environment dated 28 January 2009 concerning the conditions to be met while discharging sewage into the water or soil and regarding substances particularly harmful to the aquatic environment.

**Table 5:** Physical properties of the examined lightweight aggregate concretes.

Type of Concrete	Density (g/cm^3^)	Density Class	Apparent Density (g/cm^3^)	Absorptivity (%)	Porosity (%)
With aggregates from Mszczonów	2376	D1.6	1442	7	39.3
With aggregates from sewage sludge	2450	D1.6	1344	4	45.1

**Table 6:** Heat conductivity coefficients of lightweight aggregate-concretes.

Type of Concrete	Heat Conductivity Coefficient λ (W/mK)	
	Dry State	3% of Moisture
With aggregates from Mszczonów	0.67	0.72
With aggregates from sewage sludge	0.52	0.58

**Table 7:** List of compressive strengths of examined lightweight concrete samples.

Type of Concrete	Compressive Strength *f*’_ck_, Cube (MPa)	Concrete Class LC
With aggregates from Mszczonów	15.8	LC12/13
With aggregates from sewage sludge	11.1	LC8/9
